# Gendered impact of solid fuel use on acute respiratory infections in children in China

**DOI:** 10.1186/s12889-018-6035-z

**Published:** 2018-10-11

**Authors:** Chen Chen, Sepideh Modrek

**Affiliations:** San Francisco State University, 1600 Holloway Ave, San Francisco, 94132 CA United States

**Keywords:** Solid fuel, Indoor air pollution, Gender difference, Children, Acute respiratory infections

## Abstract

**Background:**

Indoor Air Pollution (IPA) is a serious environmental problem that can have detrimental effects on child health. In China, the major sources of indoor pollution are biomass fuel or solid cooking fuels and familial smoking. Previous studies posit that the effects of IAP on health outcomes may be worse for female children, but the empirical evidence has been mixed.

**Methods:**

In this paper we use the China Health and Nutrition Survey to examine the association of solid fuel use and paternal smoking on acute respiratory infections (ARIs) in children focusing on child gender differences. We used conditional logistic regression to examine gender differences in incidents of ARIs in the 4 weeks prior to the survey collection. We modeled gender difference by including an interaction between child gender and solid fuel use and child gender and paternal smoking. We also conducted stratified analyses by child gender.

**Results:**

When examining both genders together, female children exposed to solid fuel had an elevated risk of a ARIs, but the coefficient was not statistically significant. When using a stratified models by gender, female children had a higher risk of having ARIs in the past 4 weeks when exposed to solid fuels (OR=3.28; 95% CI 1.34-8.03) and paternal smoking (OR=2.27; 95% CI 1.08-4.77). Whereas neither exposure to solid fuel nor parental smoking had any significant influence on ARIs for male children.

**Conclusion:**

While many have hypothesized that female children may be more vulnerable to IAP, the empirical evidence has been limited. In our study we found empirical support for gender difference in the effects of solid cooking fuel use on ARIs. Gender differences in ARIs suggest that realized exposures, as opposed to ambient exposures, are likely higher for female children and are important to consider.

**Electronic supplementary material:**

The online version of this article (10.1186/s12889-018-6035-z) contains supplementary material, which is available to authorized users.

## Background

In developing countries rising air pollution is a serious environmental byproduct of rapid industrialization. Hundreds of epidemiological studies have emerged highlighting the adverse health consequences of both short-term and long-term exposure to outdoor air pollutants [1]. Likewise indoor air pollution (IAP) is another large contributor to adverse health outcomes in developing countries [2].

A large and growing literature documents the adverse health effect of IAP in developing countries, particularly for children and airway diseases and mortality [3–5]. Numerous studies indicate that exposure to the IAP can cause acute respiratory infections (ARIs) for children under 5 [6, 7] and more than half of deaths due to ARIs in children under 5 are caused by solid cooking fuels, which are the major source of IAP [8]. Meta analyses have estimated a 1.8 to 6.2 fold increase in risk of pneumonia and ARIs in children under 5 who are exposed to solid cooking fuels [9–11]. In India, solid fuel use has been associated with life threatening respiratory illness (LTRI) [12]. Evidence from a randomized controlled trial examining household air pollution on pneumonia and mortality in Guatemala found that cleaner stoves reduced the exposure to IAP by 50% [3, 4]. Interestingly, the reduction in IAP did not reduce incidences of physician-diagnosed pneumonia for children under 18 months old, but did reduce child mortality.

Despite the growing literature, there have been only a few studies conducted in China, where about 45% of households use coal as their primary fuel for cooking, and the prevalence of coal use can be as high as 79% in the rural area [13]. Shen et al. examined mortality of farmers from 1976 to 1996 in Xuanwei, China, comparing those who used smoky coal stoves to those who used smokeless coal stoves [14]. They found that smokeless stoves reduced mortality. Baumgartner et al. used PM2.5 as the measurement of IAP, particulate matter with a diameter of 2.5 micrometers, and found that biomass cooking fuel and stove maintenance are significant indicators of higher PM2.5 exposure for women, but not for children. However, PM2.5 is only one component of IAP, which includes both fine and coarse particulate matter as well as noxious chemical pollutants that can lead to health consequences.

Many studies posit that the effects of IAP on health outcomes are worse for women and female children. First, women and female children are most likely responsible for household cooking. Therefore, the probability of exposure to the solid fuel for women and female children are systematically higher. Women and female children may have higher realized exposure to IAP than ambient measures suggest because they spend more time near the source of the exposure. In addition, women and female children spend more time indoors generally, which increases the duration and accumulation of their exposure to the IAP. Moreover, the vast majority of respiratory infections are treated at home where gender norms may lead to differential treatment and prioritization.

Despite these hypothesized differences, few studies systematically reported gender differences. Studies that have looked at gender differences often examine more severe health outcomes such as ARI/pneumonia, LTRI or mortality. The objective of our current study is to examine a) whether exposure to solid fuel is associated with a milder indicator of respiratory infections, having a fever, sore throat or cough during the 4 weeks prior to the survey collection, b)a broader age range than previous studies, including all children under age 18 and c) potential gender differences in detail.

## Data

The data was obtained from the China Health and Nutrition Survey (CHNS), an international collaborative project by Carolina Population Center at the University of North Carolina at Chapel Hill and the National Institute for Nutrition and Health at the Chinese Center for Disease Control and Prevention. CHNS used a multistage random cluster process to draw a sample of panel households. Nine provinces were selected as the main sample frame. Within each province four counties (one low-income, two middle-income, and one high-income) and two cities (the capital city and a low-income city) were chosen. Next, within the four counties and two cities the primary sampling units (PSU) were selected. Twenty households were empaneled and surveyed within each PSU. Households that move were not followed unless they are in the survey PSU.

In each wave the entire survey was collected over a 7-day period.^1^ In this paper, we examined children under 18 in the 2004, 2006, 2009, and 2011 survey waves. Our sample included 1940 children under age 18 [15].

### Outcome variable

The outcome variable of interest was if the child had an acute respiratory infection in the past four weeks as measured by reporting fever, cough, or sore throat. In each survey wave, all children over the age of 10 were asked, “[Did you have any of these symptoms [fever, cough, or sore throat] during the past 4 weeks (including today)?]”. For children under 10, their parents responded to the same question on their behalf. If a child or parent affirmed having had a fever, cough, sore throat, then we designated the child as having had an acute respiratory infection.

#### Independent variables

The independent variables of interest were the presence of indoor air pollution from the use of solid fuel for cooking and indoor smoking. We created an indicator for household solid fuel use, “cook with solid fuel”, if the household used coal, wood, or charcoal for cooking. We followed the previous literature and considered the use of electricity, kerosene, liquefied or natural gas as clean cooking fuels [16]. In each survey year, the usage of solid fuel were asked again, which allowed us to capture and update any changes in solid fuel use in the household. Another major source of IAP is smoke from cigarettes. In each survey wave, all fathers were asked “[Have you ever smoked cigarettes?]”. Based on this question we created a variable of paternal smoking. We also explored maternal smoking, but there were very few cases in our sample, so we focused on fathers’ smoking behavior.

## Methods

Our main analyses examined whether exposure to solid cooking fuels was associated with an acute respiratory infection. We estimated the association between exposure to the solid fuel and ARIs by using Conditional Fixed Effect Logistic Regression model. For each child, i, in community, c, in survey wave, t, we estimated the following equation:

1 $$\begin{array}{*{20}l} {}Y_{ict}&=Z\beta_{0}+\beta_{1}solid fuel_{ict}+\beta_{2}father smoke_{ict}+\beta_{3}X_{ict}\\ &\quad+t_{t}+a_{c}+u_{ict} \end{array} $$

where *Y*_*ict*_ is an indicator for having an ARI in the last four weeks, *solidfuel*_*ict*_ represents the measure of exposure to solid fuels for that child in that survey; *X*_*ict*_ includes a vector of individual and household characteristics for each child; *t*_*t*_ are survey year indicator variables. Our composite error term is *a*_*c*_+*u*_*ict*_, which we modeled in two ways. We modeled common community-level effects, *a*_*c*_, using either Random-Effects or Fixed-Effects models. Random-effects models assume that community-level effects are uncorrelated with the other covariates in the model. Random-effects models are more efficient, but may be inconsistent. Fixed-effects models assume that community-level effects are correlated with the other covariates in the model, and are less efficient but more likely to be consistent. These two model specifications for community-level effects were compared using a Hausman test (see Additional file 1: Table S1). Results suggested that there is significant difference between Fixed-Effect models and Random-Effect models. Thus, we chose the more consistent model, Fixed-Effect, to reduce the potential for unobservable bias. The inclusion of community-level Fixed-Effects also accounted for common local seasonal infections. The survey year (time) indicators controlled for secular changes in seasonal infections. The same model were applied in the stratified analyses by child gender.

After estimating the logistic regression models detailed above, we used the margins command in STATA 14 [17] to predict the probability of ARI for both exposed and unexposed groups. The difference in the predicted probabilities is the marginal effects, also known as marginal standardization, which we report [18]. Estimates of the marginal effects capture the difference in probability of ARI had we been able to force all of the study population to change from unexposed to exposed for either solid fuel use or paternal smoking.

In the main estimation, we used all observations including repeat observation for children over different survey waves assuming that ARIs are independent events. However, we conducted several supplemental analyses to ensure our results were robust. First, we reestimated the main model specification above, using only the first observation for each child to avoid the potential for individual-level clustering over survey wave. Second, we reestimate the equation for a subsample of children for whom we have some information on their chores in the past week. For this subset of children, 30% of female children reported engaging in food preparation whereas 18% of male children reported engaging in food preparation in the past week. While activities within food preparation may be still be gendered in terms of tasks (i.e. direct cooking and exposure to solid fuel versus chopping) and time spent, food preparation may be a confounder. We reestimated the main model specification and include an additional indicator variable for reporting any food preparation chore in the past week.

## Results

Table 1 presents the summary statistics for our sample. In the full sample, 45% children are female. ARIs are common with 14.99% of female children and 12.25% of male children reporting an incident in the past four weeks. On average, 41.95% of households use solid fuel as their primary cooking fuel, and 65.25% of fathers smoke. In our sample, 63.3% of fathers of male children smoke compared to 67.6% of fathers of female children. In households with a male child, 43.5% use a solid cooking fuel compared to 40.1% of households with female children.

**Table 1:** Summary statistics and standardized difference by child gender

	Total (*N*=1940)	Male (*N*=1053)	Female (*N*=877)	Standardized difference by child gender
Child characteristics				
Fever, cough, soar throat (%)	13.51	12.25	14.99	0.08
Female (%)	45.72			
Age	11.56	11.68	11.41	0.06
Mother’s education level				
Graduated from primary (%)	20.62	20.42	20.86	0.01
Middle school degree (%)	40.41	41.03	39.68	0.03
High school degree (%)	18.92	19.09	18.71	0.01
College degree or above (%)	4.79	3.13	6.76	0.17
Household wealth				
Household income per capita	7051.90	7097.35	6997.94	0.01
Rooms	4.87	5.02	4.69	0.11
House size (square meters)	118.58	124.05	112.09	0.15
Permeability				
Permeable roof (%)	28.40	27.82	29.08	0.03
Indoor Air Pollution (IAP)				
Use solid cooking fuel (%)	41.95	43.49	40.13	0.07
Father’s smoking(%)	65.25	63.25	67.64	0.09

Note: Absolute standardized differences in means by gender are presented. Values above 0.1 suggest systematic difference by gender

There are some systematic differences across genders in terms of household socioeconomic status. For example, percentage of mother having a college degree is higher for mother’s of daughters. In terms of the house conditions and ventilation, households who have sons have more rooms and larger house size than households who have daughters (100 sq. meters larger). Additionally, households having sons have lower roof permeability which also indicates that family with sons have better economic status than families with daughters. Compared to households with female children, on average, households having male children have a household income 100 yuan higher per capita [19]. Of these differences maternal education, number of room and household size have a moderately large standardized difference across genders.

Table 2 presents odds ratios for the effect of IAP on ARIs in three models. The first model includes both male and female children together where the gender differences are tested with interaction terms between child gender and solid cooking fuel and child gender and father’s smoking status. The odds ratio of the interaction effect between solid fuel use and child gender female is elevated but not statistically significant (OR =1.55, 95% CI 0.88-2.76). Likewise, the odds ratio of the interaction effect between paternal smoking and child gender female is elevated but again not statistically significant (OR =1.51, 95% CI 0.82-2.76).

**Table 2:** Association between solid sooking fuel and scute respiratory infections by child gender, odd ratios

	Total		Male		Female	
	OR	95%CI	OR	95%CI	OR	95%CI
IAP						
Solid cooking fuel	1.29	[0.72-2.33]	0.95	[0.44-2.04]	3.28**	[1.34-8.03]
Father smokes	0.98	[0.60-1.59]	0.80	[0.44-1.46]	2.27*	[1.08-4.77]
Solid x Smoke	0.92	[0.50-1.69]	1.45	[0.60-3.52]	0.49	[0.18-1.30]
Solid x Female	1.55	[0.87-2.75]				
Smoke x Female	1.50	[0.82-2.75]				
Child characteristics						
Female	0.76	[0.42-1.36]				
Age	0.77***	[0.67-0.89]	0.75**	[0.60-0.92]	0.75*	[0.60-0.93]
Age-squared	1.00*	[1.00-1.01]	1.01*	[1.00-1.02]	1.01*	[1.00-1.02]
Mother’s education level						
Middle school Degree	0.76	[0.54-1.09]	0.64	[0.36-1.12]	0.81	[0.48-1.37]
High school Degree	0.81	[0.49-1.32]	0.63	[0.29-1.36]	0.71	[0.33-1.50]
College degree and above	0.65	[0.28-1.52]	0.34	[0.07-1.60]	0.78	[0.23-2.65]
Wealth						
Housesize	1.00	[1.00-1.00]	1.00	[0.99-1.00]	1.00	[0.99-1.00]
Number of rooms	0.94	[0.88-1.01]	0.95	[0.84-1.07]	0.96	[0.86-1.06]
Log(Household net income per capita)	1.01	[0.87-1.18]	0.87	[0.69-1.09]	1.09	[0.84-1.40]
Permeability						
Roof Permeable	0.79	[0.53-1.17]	0.49*	[0.26-0.92]	1.09	[0.60-1.97]
Community fixed effect	Yes		Yes		Yes	
Time fixed effect	Yes		Yes		Yes	
Observation	1940		828		697	

* *p* <0.05, ** *p* <0.01, *** *p* <0.001

In the second and third columns of Table 2 we present sex stratified models. The second column presents results for male children and the third presents results for female children separately. For male children only age and age squared have a significant negative effect on ARIs with increasing age leading to a decrease in the risk of having ARIs. For male children having a permeable roof decreases the likelihood of having an ARI (OR =0.495; 95% CI 0.27-0.92). We find no association for either solid cooking fuel use or paternal smoking and ARIs for male children.

While exposure to solid fuel is essentially not related to ARI in male children, the odds ratio of exposure to solid cooking fuel for female children is elevated at 3.28 (95% CI 1.34-8.03) and statistically significant. Likewise, another measurement of IAP, father’s smoking is again related to ARIs in female children but not male children. Daughters exposed to fathers’ smoking have 2.27 (95% CI 1.08-4.78) times higher likelihood of having ARIs in the last 4 weeks. These elevated odds ratios can be translated to average marginal effects, the difference in risk for ARI for exposed compared to unexposed female children. The estimates suggest that female children exposed to solid fuel have a 16.8% increase in probability of having an acute respiratory infection in the past 4 weeks, and female children exposed to paternal smoking have a 12.6% increase in probability of having an upper respiratory infection in the past 4 weeks (see Additional file 1: Table S2).

Since ARI are acute events that can happen at anytime, the models above treats each ARI as an independent event. However, since we have repeated observations on the same individuals in our sample, we also conducted analyses including only the first observation per individual to avoid any individual-level clustering. Results suggest that female children exposed to solid cooking fuels are more likely to have an ARI, but the results are only marginally significant (see Additional file 1: Table S3; OR = 4.02, *P*-val =0.07). Male children do not have an elevated risk of ARIs. These results are in the same direction as those presented in Table 2, but are likely underpowered. All other factors including age and wealth factors are consistent across genders with our main results.

We also present analyses for a subsample of children where we were able to account for any food preparation activity in the week prior to survey enumeration in Additional file 1: Table S4. We find that participation in food preparation is associated with higher risk of ARI for both genders, though it is not statistically significant. Furthermore, including this control strengthens the association of ARI for female children who are exposure to the solid cooking fuel. For female children exposed to solid cooking fuel their risk of ARIs remains elevated and statistically significant (see Additional file 1: Table S4; OR =3.75, 95% CI 1.05-13.40).

## Discussion

We examined the association between exposure to solid fuel and acute respiratory diseases for children under 18, especially the differences between the males and females. In initial analyses with exposure and gender interactions, there was evidence of an elevated risk of ARI for exposure to solid fuels for female children, but the coefficients were not statistically significant. In stratified models, females who are exposed to solid cooking fuel and father’s smoking had significantly higher risk of having ARIs in the past four weeks.

This study made two contributions. First we focused on gender difference in detail. While previous studies have examined gender interactions they may not have found differences because they did not stratify the model which may better accounts for multiple gender differences in contracting ARIs [12]. Second most existing literature focuses on severe or fatal respiratory diseases or mortality rate as their health outcome [11]. These types of outcomes capture more serious disease but cannot capture more common infections. Looking at more severe disease may also lead to measurement issues because parents may systematically take male children to the hospital more often than their female children for ARIs. Using child reported fever, cough, sore throat may capture minor respiratory infections but perhaps allows to more clearly observe the gender difference.

Nonetheless there are several notable limitations in this study. First, we used minor illnesses as a proxy for ARI and there is a potential for misclassification, since other mild illness, such as ear infections which also lead to fevers, are included in our outcome measure. Therefore the magnitude of ARIs may be overestimated in our results. However, since the outcome measurement is the same for households with and without solid fuel use and for male and female children, the systematic difference we document by solid fuel use and gender remain valid. Second, we excluded 9705 children because of missing data covariates, which limited the sample size substantially. Finally, while we try to account for food preparation activities for a subsample in additional analyses, we have no indicator of the time that children spend in kitchen while their parents are cooking or the number of window in the kitchen or where fathers smoke (indoors or outdoors), all of which would allow us to better isolate exposure to IAP [6, 10, 20, 21].

We presume that many of the gender differences are based on location and time spent indoors. In China, mothers are responsible for cooking and female children are more likely to stay with their mothers while cooking. Therefore young female children may be exposed to solid fuels because they collocate with mothers and may be indoors more. Compared to female children, male children spend more time outside so that the exposure to the indoor air pollution caused by father’s smoking and solid fuel may be limited and mitigated. According to past studies, the gender difference of time spent in kitchen and living area is relatively small before age 6, but increase dramatically later between ages 6-19 [21]. This is supported in our data as well. Since other studies often only examine children under age 6, they may not observe gender difference that become more salient as female children age and spend more time in areas with higher IAP concentration. Our study includes children up to age 18 and therefore our results capture female children at older ages where their exposure to IAP may be systematically higher.

Finally, since solid fuel and roof permeability have a significant influence on all household members, but particularly female children, policy makers can focus on policies to improve the cooking environment and/or improve the permeability of housing material for the entire household [3, 4, 14]. For example, several stove improvement experiments have shown that drastic declines in IAP are possible [9, 14, 20, 22]. Moreover, informing parents of the gender differences in ARI may lead to some changes in delegation of household tasks or mitigating behaviors. However, since many families still have strong son preferences, improving the overall living environment may be a more effective strategy.

## Conclusion

While many have hypothesized that female children may be more vulnerable to IAP, the empirical evidence has been limited. In our study we make several methodological contributions such as a) using mother and child reported cases of ARI, b) having a broader definition of ARI, c) expanding the age ranges of included children, and d) stratify our analyses by gender. In doing so we find empirical support for gender differences in the effects of IAP caused by solid cooking fuel on ARIs. Increased exposure to solid cooking fuel increases the potential risk of having fever, cough, and sore throat for female children. Moreover, father’s smoking also has a similar effect as another important source of indoor air pollution. Parents should be more aware of the health consequence of using solid cooking fuel and try to limit their children’s exposures, especially the exposure of female children.

## Additional file

Additional file 1 Supplementary Tables. (PDF 35 kb)

## Abbreviations

ARIs: Acute respiratory infections
FE: Fixed effect
IAP: Indoor air pollution
PSU: Primary sampling unit

## Footnotes

1 This remarkable timing is important to note because once we account for the timing of each survey wave and location of the households, we do not need to account for seasonal variation within survey waves.
