# Supplementary material for: Gendered impact of solid fuel use on acute respiratory infections in children in China
Source: BMC Public Health. 2018 Oct 11;18:1170. doi: 10.1186/s12889-018-6035-z (PMC6182818; doi:10.1186/s12889-018-6035-z)
Supplement: Supplementary file 1 — Supplementary Tables. (PDF 35 kb) [file 12889_2018_6035_MOESM1_ESM.pdf]

Table Supplemental1: Comparing Fixed Effect and Random Effect Model Specifications

	Coefficients<br>Fixed Effect	Random Effect	Difference	S.E.
**IAP**				
Solid Cooking Fuel	0.25	0.05	0.20	0.12
Father smokes	-0.01	-0.06	0.04	0.08
solid x smoke	-0.08	-0.19	0.11	0.10
Solid x Female	0.44	0.42	0.01	0.09
Smoke x Female	0.41	0.46	-0.05	0.97
**Child Characteristics**				
Female	-0.27	-0.26	-0.01	0.11
Age	-0.25	-0.26	0.01	0.02
Age-squared	0.84E-02	0.97E-02	-0.13E-02	0.13E-02
**Mother's Education Level**				
Middle school Degree	-0.26	-0.22	-0.03	0.07
High school Degree	-0.20	-0.06	-0.14	0.13
College degree and above	-0.42	-0.07	-0.34	0.25
Wealth				
Housesize	0.22E-02	0.14E-02	0.8E-03	0.5E-03
Number of rooms	-0.05	-0.04	-0.59E-02	0.01
Log (Household net income per capita)	0.01	0.06	-0.04	0.03
**Permeability**				
Roof Permeable	-0.23	-0.13	-0.09	0.11

b= consistent under Ho and Ha;
obtained from xtlogit
B= inconsistent under Ha,
efficient under Ho;
obtained from xtlogit

Test: Ho: difference in coefficients not systematic

chi2(18) = (b-B)'[(V_b-V_B)^(-1)](b-B)

= 29.45

Prob>chi2 = 0.04

Table Supplemental 2: Predicted Probabilities and Marginal Effects of Acute Respiratory Infections From Logistic Regressions, Marginal Standardization Method

Predicted Probabilities of ARI

	Female	Male
Solid Fuel Exposure		
No	45.29%	2.99%
Yes	62.10%	3.53%
Marginal Effects (Difference)	16.81%	0.54%
Paternal Smoking		
No	43.43%	3.29%
Yes	56.00%	3.24%
Marginal Effects (Difference)	12.57%	-0.06%

Table Supplemental3: Association between Solid Cooking Fuel and First Acute Respiratory Infection by Child Gender, Odd Ratios

	Total		Male		Female	
	OR	95%CI	OR	95%CI	OR	95%CI
**IAP**						
Solid Cooking Fuel	1.84	[0.73-4.60]	1.60	[0.43-5.91]	4.02	[0.87-18.4]
Father smokes	1.60	[0.73-3.49]	1.42	[0.52-3.83]	2.06	[0.53-7.92]
solid x smoke	0.87	[0.35-2.17]	1.23	[0.31-4.87]	0.38	[0.07-1.92]
solid x female	1.32	[0.60-2.92]				
smoke x female	0.92	[0.60-2.92]				
**Child Characteristics**						
Female	0.98	[0.40-2.41]				
Age	0.82*	[0.69-0.98]	0.70*	[0.51-0.96]	0.80	[0.51-0.96]
Age-squared	1.00	[0.99-1.01	1.01	[0.99-1.02]	1.00	[0.99-1.02]
**Mother's Education Level**						
Middle school Degree	1.02	[0.63-1.65]	0.97	[0.44-2.15]	0.99	[0.47-2.06]
High school Degree	0.91	[0.46-1.80]	0.49	[0.14-1.74]	1.27	[0.45-3.53]
College degree and above	1.23	[0.37-4.04]	0.55	[0.071-4.23]	1.27	[0.21-7.50]
**Wealth**						
Housesize	1.00	[0.99-1.00]	0.99	[0.99-1.00]	1.00	[0.99-1.00]
Number of rooms	0.98	[0.88-1.09]	0.95	[0.79-1.14]	1.04	[0.90-1.20]
Log(Household net income per capita)	1.06	[0.84-1.34]	1.00	[0.72-1.04]	0.98	[0.65-1.48]
**Permeability**						
Roof Permeable	0.78	[0.45-1.33]	0.35*	[0.14-0.82]	1.64	[0.68-3.96]
**Community Fixed Effect**	Yes		Yes		Yes	
**Time Fixed Effect**	Yes		Yes		Yes	
**Observation**	775		287		258	
* $p<0.05$	** $p<0.01$		*** $p<0.001$			

Note: The total number of male and female children do not add up to 775 because certain communities were dropped from the analyses because there was no variation in the ARI outcome by gender.

Table Supplemental 4: Association between Solid Cooking Fuel and ARIs with Food Preparation Controls, by Child Gender, Odd Ratios

	Total		Male		Female	
	OR	95%CI	OR	95%CI	OR	95%CI
**IAP**						
Solid Cooking Fuel	1.12	[0.45-2.77]	1.12	[0.29-4.31]	3.75*	[1.05-13.40]
Father smokes	1.00	[0.48-2.06]	0.98	[0.37-2.63]	2.64	[0.96-7.25]
solid x smoke	0.62	[0.24-1.55]	0.92	[0.22-3.89]	0.44	[0.10-2.00]
solid x female	2.40	[0.99-5.82]				
smoke x female	1.87	[0.77-4.53]				
**Child Characteristics**						
Female	0.48	[0.20-1.17]				
Age	0.71	[0.42-1.19]	0.79	[0.33-1.92]	0.42*	[0.17-0.99]
Age-squared	1.01	0.99-1.03]	1.00	[0.97-1.04]	1.03	[0.99-1.06]
Food Preparation	1.35	[0.78-2.34]	1.14	[0.42-3.05]	2.00	[0.84-4.76]
**Mother's Education Level**						
Middle school Degree	0.55*	[0.33-0.93]	0.51	[0.21-1.25]	0.35*	[0.153-0.838]
High school Degree	0.57	[0.27-1.23]	0.54	[0.16-1.74]]	0.50	[0.14-1.74]
College degree and above	0.24	[0.04-1.20]	0.30	[0.01-5.26]	0.32	[0.02-3.88]
**Wealth**						
Housesize	1.00	[0.99-1.00]	0.99	[0.99-1.00]	1.00*	[1.00-1.01]
Number of rooms	0.94	[0.85-1.04]	1.01	[0.84-1.23]	0.88	[0.75-1.03]
Log(Household net income per capita)	1.07	[0.85-1.35]	0.85	[0.56-1.28]	1.08	[0.73-1.60]
Permeability						
Roof Permeable	1.01	[0.58-1.77]	0.51*	[0.19-1.34]	2.56	[0.99-6.63]
**Community Fixed Effect**	Yes		Yes		Yes	
**Time Fixed Effect**	Yes		Yes		Yes	
**Observation**	783		273		287	
* p<0.05	** p<0.01		*** p<0.001			

Note: The total number of male and female children do not add up to 783 because certain communities were dropped from the analyses because there was no variation in the ARI outcome by gender.
